# Supplementary material for: Study on the Antiviral Activities and Hemagglutinin-Based Molecular Mechanism of Novel Chlorogenin 3-O-β-Chacotrioside Derivatives against H5N1 Subtype Viruses
Source: Viruses. 2020 Mar 11;12(3):304. doi: 10.3390/v12030304 (PMC7150989; doi:10.3390/v12030304)
Supplement: Supplementary file 1 [file viruses-12-00304-s001.pdf]

Supporting Information

Table S1. Primers for site-directed HA plasmids

Primer name	Primer sequence(5--3′)
A/Thailand/kan353/2004-I48A-F	CATGCCCAAGACGCACTGGAAAAGACAC
A/Thailand/kan353/2004-I48A-R	GTGTCTTTTCCAGTGCGTCTTGGGCATG
A/Thailand/kan353/2004-L329F-F	CAGATTAGTCTTTGCGACTGGGCTC
A/Thailand/kan353/2004-L329F-R	GAGCCCAGTCGCAAAGACTAATCTG
A/Thailand/kan353/2004-T331A-F	AGTCCTTGCGGCTGGGCTCAGAAAT
A/Thailand/kan353/2004-T331A-R	ATTTCTGAGCCCAGCCGCAAGGACT
A/Thailand/kan353/2004-W367R-F	GGTAGATGGTCGGTATGGGTACCAC
A/Thailand/kan353/2004-W367R-R	GTGGTACCCATACCGACCATCTACC
A/Thailand/kan353/2004-T387A-F	GACAAAGAATCCGCTCAAAAGGCAATAG
A/Thailand/kan353/2004-T387A-R	CTATTGCCTTTTGAGCGGATTCTTTGTC
A/Thailand/kan353/2004-V394A-F	GCAATAGATGGAGCCACCAATAAGGTC
A/Thailand/kan353/2004-V394A-R	GACCTTATTGGTGGCTCCATCTATTGC
A/Thailand/kan353/2004-T395A-F	AGATGGAGTCGCCAATAAGGTCAAC
A/Thailand/kan353/2004-T395A-R	GTTGACCTTATTGGCGACTCCATCT

Table S2. The titrations of H5N1 subtype influenza virus.

Number	Virus dilutions									
	10^{-1}	10^{-2}	10^{-3}	10^{-4}	10^{-5}	10^{-6}	10^{-7}	10^{-8}	10^{-9}	10^{-10}
1	++++	++++	++++	++++	++++	++++	++++	−	−	−
2	++++	++++	++++	++++	++++	−	−	−	−	−
3	++++	++++	++++	++++	++++	++++	−	−	−	−
4	++++	++++	++++	++++	++++	−	++++	−	−	−

5	++++	++++	++++	++++	++++	++++	–	–	–	–
6	++++	++++	++++	++++	++++	–	–	++++	–	–

Note:"–" indicated no cytopathic changes; "+" indicated that 25% of cells occurred pathological changes; "++" represented 50% cytopathic changes; "+++" represented 75% cytopathic changes; "++++" represented 100% cytopathic changes.

Table S3. The inhibition rates of the derivatives against H5N1 virus (%)（$\bar{x}$± S, n=3）

Concentration （μM）	Inhibition rates to H5N1 virus (%)			
	Control group	chlorogenin 3-O-β-chacotrioside derivatives		
		UA-Nu-ph-5	XC-27-1	XC-27-2
0.39	0	0	27.54±1.11	31.94±4.33
0.78	0	10.75±2.01	31.73±6.04	38.61±0.68
1.56	0	10.78±3.11	37.01±4.82	41.49±2.01
3.13	0	16.51±6.34	40.85±8.91	47.09±5.94
6.25	0	35.02±10.81	46.13±10.59	55.56±3.97
12.5	0	47.06±1.63	55.30±3.74	62.21±0.99
25	0	61.69±5.4	75.06±10.43	70.98±4.31
50	0	65.30±4.73	85.71±5.06	96.85±1.51
100	0	79.10±3.27	—	—

The inhibition rate of the derivatives are means of three independent experiments (n= 3, mean ± S.E.M.).

Table S4.The infectivity of H5N1 and VSVG pseudovirus with 10-fold diluted to MDCK cells（$\bar{x}$± S, n=3）

Tested index	Groups		
	Vehicle control	H5N1*pseudovirus*	VSVG *pseudovirus*
Relative Light Unit	159±62	26489122±5651220**	697528±2749**

The infectivity was measured by luciferase assay method. The RLU of pseudovirus with10-fold diluted are means of three independent experiments (n=3, mean ±S.E.M.). Difference was considered statistically significant when*p<0.05 and **p<0.01vs vehicle control group.

Figure S1. The infectivity ability of *pseudovirus* to MDCK cells

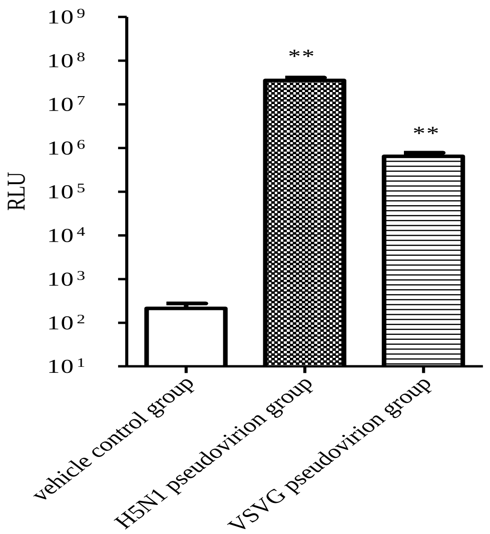

Figure S1. The infectivity ability of *pseudovirus* to MDCK cells

Table S5. Inhibition rates of derivatives against H5N1 pseudoviruses.

Concentration (μM)	The inhibitory rates of derivatives (%)			
	XC-27-1		XC-27-2	
	H5N1*pseudovirus* group	VSVG *pseudovirus* group	H5N1*pseudovirus* group	H5N1*pseudovirus* group
1.56	33.59±5.25	-0.74±6.43	26.83±9.96	2.62±4.57
3.13	38±3.24	-1.52±4.89	30.10±12.17	-3.39±4.64
6.25	40.69±10.88	-4.3±3.47	35.80±12.13	0.17±5.78
12.5	48.95±11.65	3.54±4.16	46.30±6.37	1.54±13.89
25	60.68±3.73	0.83±6.72	47.43±13.16	0.46±0.94
50	64.81±2.96	3.97±5.20	64.88±12.50	0.34±6.12
100	78.01±2.16	4.80±4.97	90.53±2.35	6.84±5.83
200	95.32±3.6	4.75±3.18	95.98±0.68	6.52±2.16

The inhibition rate after treatment with derivatives at two-fold serial diluted concentrations for 48h are means of three independent experiments (n=3, mean ± S.E.M). Each group set 6 parallel wells. Difference was considered statistically significant when * $p<0.05$ and ** $p<0.01$ vsVSVG *pseudovirus* group.

Table S6. NA inhibitory rate of compounds against H5N1 pseudotyped viruses ($\bar{x}$ ± S, n=3)

Concentration	The neuraminidase inhibitory rate (%)

（μM）	XC-27-1	XC-27-2
400	24.46±8.38	32.02±10.02
200	7.07±9.83	15.57±2.97
100	6.45±14.87	13.34±4.06
50	5.37±14.44	10.51±16.46
25	-6.95±12.67	2.89±8.39
12.5	0.18±8.13	5.75±4.41
6.25	6.57±6.89	1.09±2.99

Figure S2. Observation of viral plaques formation after the infected cells was dealt with the derivative XC-27-1

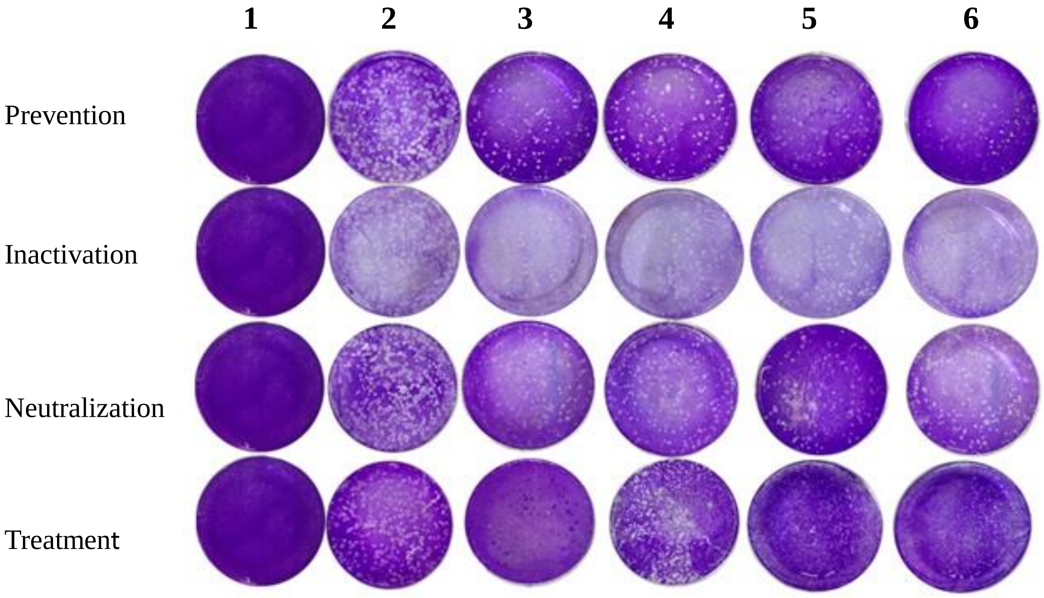

Figure S2. Observation of viral plaques formation after the infected cells was dealt with the derivative XC-27-1 at four different patterns. Using H5N1 virus as a challenge virus, the dealt manners from the 1st to the 4th of column were prevention, inactivation, neutralization and treatment in turn. The wells of the 1st column were the uninfected cell control (vehicle control), the wells of the 2nd column were the infected cell control with the challenge virus dose (negative control), and the wells of the 3rd column were the infected cells treated with ribavirin (positive control). The wells of last three columns were the infected cells dealt with 10.0μM, 20.0μM and 40.0 μM XC-27-1, respectively.

Figure S3. Observation of viral plaques formation after the infected cells was dealt with the derivative XC-27-2

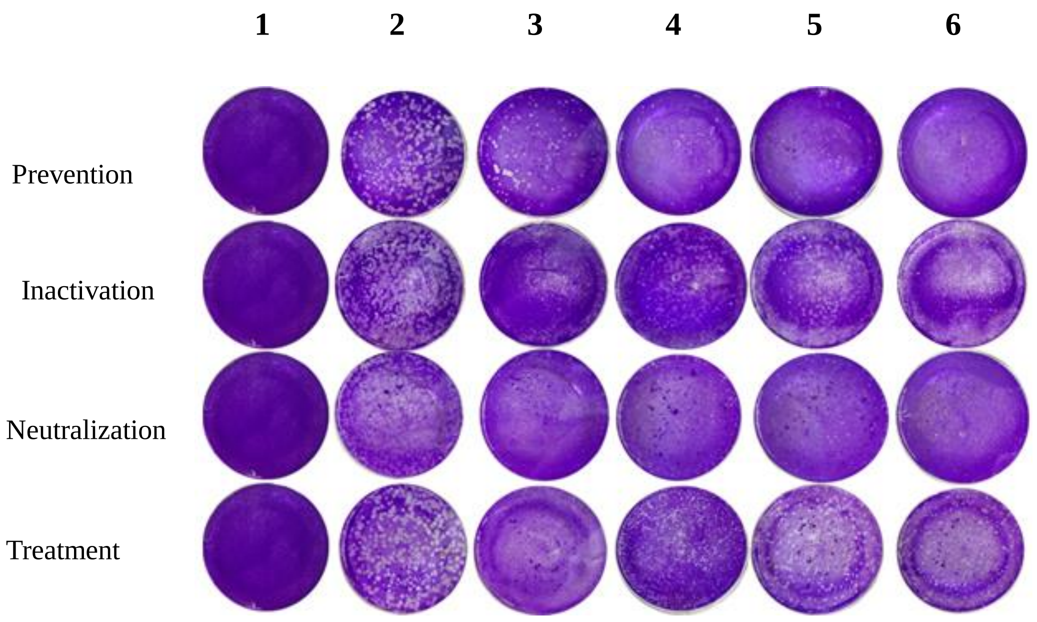

Figure S3. Observation of viral plaques formation after the infected cells was dealt with the derivative XC-27-2 at four different patterns. Using H5N1 virus as a challenge virus, the dealt manners from the 1st to the 4th of column correspond to prevention, inactivation, neutralization and treatment. The wells of the 1st column were vehicle control, the wells of the 2nd column were negative control, and the wells of the 3rd column were positive control. The wells of last three columnswere the infected cells dealt with 10.0μM, 20.0μM and 40.0 μM XC-27-2, respectively.

Figure S4. Observation of viral plaques formation after the infected cells was dealt with the derivative UA-Nu-ph-5

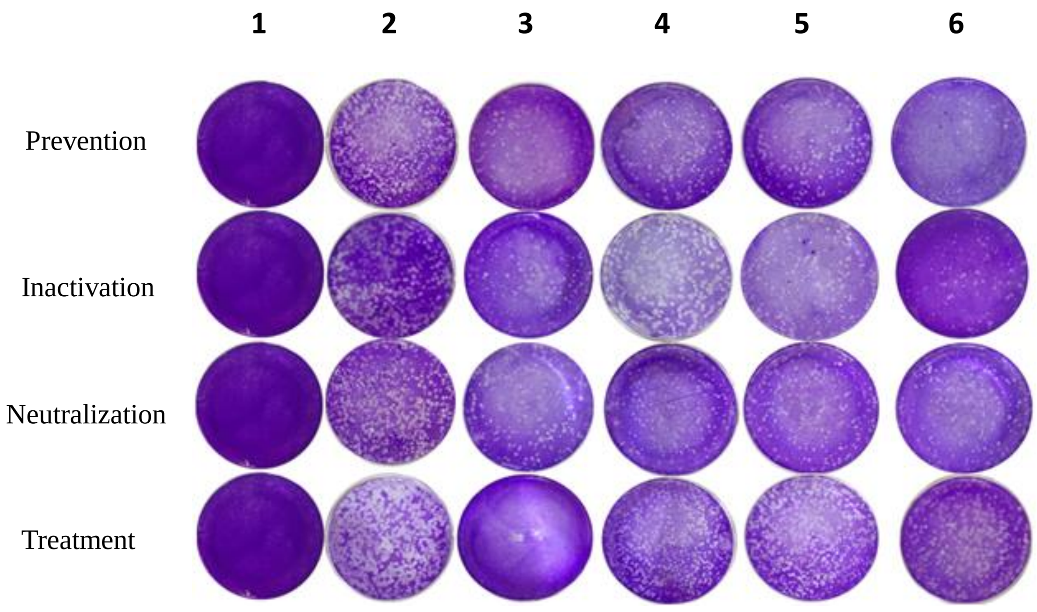

Figure S4. Observation of viral plaques formation after the infected cells was dealt with the derivative UA-Nu-ph-5 at four different patterns. Using H5N1 virus as a challenge virus, the dealt manners from the 1st to the 4th of column correspond to prevention, inactivation, neutralization and treatment. The wells of the 1st column were vehicle control, the wells of the 2nd column were negative control, and the wells of the 3rd column were positive control. The wells of last three columns were the infected cells dealt with 10.0μM, 20.0μM and 40.0 μM UA-Nu-ph-5, respectively.

Figure S5. The association-dissociation curves of the binding interaction among the derivatives and HA

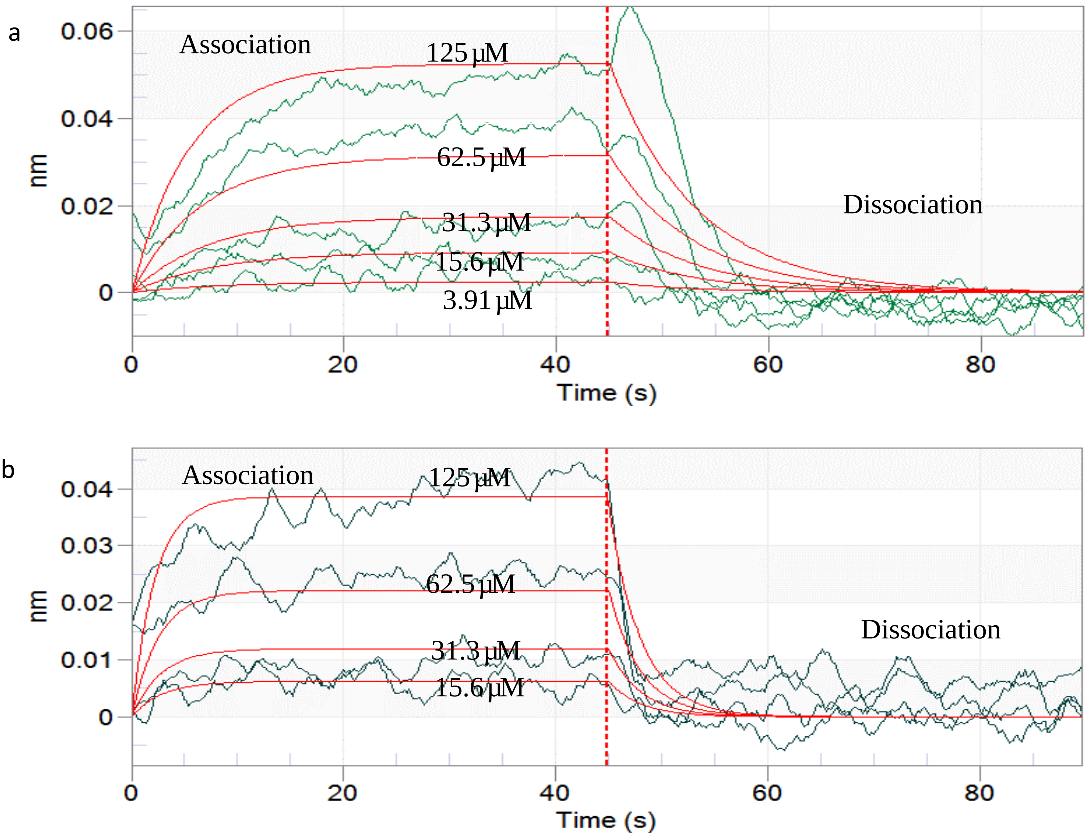

FigureS5.The association-dissociation curves of the binding interaction among the derivatives and HA. Fig19-a represents the curves of association and dissociation of XC-27-1 at varying concentrations: 125μM, 62.5μM, 31.3μM, 15.6μM, 3.91μM. Fig19-b represents the curves of association and dissociation of XC-27-2 at varying concentrations: 125μM, 62.5μM, 31.3μM, 15.6μM. The experiments were repeated at least three times.

Figure S6. Structural comparison for potential binding sites between HA-compound complex with HA-CT149 antibody complex.

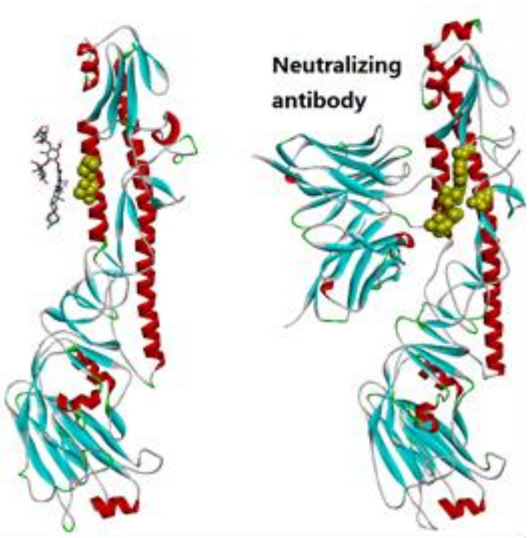

Figure S6. Structural comparison for potential binding sites between HA-compound complex with HA-CT149 antibody complex. Only monomer of HA crystal structure are shown in the figure. All of the available HA sequences and highly homologous HA crystal structure were retrieved from NCBI and PDB databases, and structure figure was drawn using Discovery Studio program. The yellow balls of left figure represent the key residues for binding of compound to HA. The yellow balls of right figure represent the key residues for binding of CT149 antibody to HA.

Figure S7. Sequence comparison of A/Thailand/Kan353/2004-HA plasmid used in this experiment with A/Thailand/Kan353/2004-HA gene retrieved from NCBI databases. Among them, Query represents the plasmid used in this study, and Sbjct represents A/Thailand/Kan353/2004-HA plasmid retrieved from NCBI databases.

```
Query  1     ATGGAGAAAATAGTGCTTCTTTTTGCAATAGTCAGTCTTGTTAAAAGTGATCAGATTTGC  60

||||||||||||||||||||||||||||||||||||||||||||||||||||||||||||

Sbjct  9     ATGGAGAAAATAGTGCTTCTTTTTGCAATAGTCAGTCTTGTTAAAAGTGATCAGATTTGC  68


Query  61    ATTGGTTACCATGCAAACAACTCGACAGAGCAGGTTGACACAATAATGGAAAAGAACGTT  120

||||||||||||||||||||||||||||||||||||||||||||||||||||||||||||

Sbjct  69    ATTGGTTACCATGCAAACAACTCGACAGAGCAGGTTGACACAATAATGGAAAAGAACGTT  128


Query  121   ACTGTTACACATGCCCAAGACATACTGGAAAAGACACACAACGGGAAGCTCTGCGATCTA  180

||||||||||||||||||||||||||||||||||||||||||||||||||||||||||||

Sbjct  129   ACTGTTACACATGCCCAAGACATACTGGAAAAGACACACAACGGGAAGCTCTGCGATCTA  188
```

```
Query  181   GATGGAGTGAAGCCTCTAATTTTGAGAGATTGTAGTGTAGCTGGATGGCTCCTCGGAAAC  240
             ||||||||||||||||||||||||||||||||||||||||||||||||||||||||||||
Sbjct  189   GATGGAGTGAAGCCTCTAATTTTGAGAGATTGTAGTGTAGCTGGATGGCTCCTCGGAAAC  248

Query  241   CCAATGTGTGACGAATTCATCAATGTGCCGGAATGGTCCTACATAGTGGAGAAGGCCAAC  300
             |||||||||||||||||||||||||||||||||||||||||||||||||||||||||||
Sbjct  249   CCAATGTGTGACGAATTCATCAATGTGCCGGAATGGTCCTACATAGTGGAGAAGGCCAAT  308

Query  301   CCAGTCAATGACCTCTGTTACCCAGGGGATTTCAATGACTATGAAGAATTGAAACACCTA  360
             ||||||||||||||||||||||||||||||||||||||||||||||||||||||||||||
Sbjct  309   CCAGTCAATGACCTCTGTTACCCAGGGGATTTCAATGACTATGAAGAATTGAAACACCTA  368

Query  361   TTGAGCAGAATAAACCATTTTGAGAAAATTCAGATCATCCCCAAAAGTTCTTGGTCCAGT  420
             ||||||||||||||||||||||||||||||||||||||||||||||||||||||||||||
Sbjct  369   TTGAGCAGAATAAACCATTTTGAGAAAATTCAGATCATCCCCAAAAGTTCTTGGTCCAGT  428

Query  421   CATGAAGCCTCATTAGGGGTGAGCTCAGCATGTCCATACCAGGGAAAGTCCTCCTTTTTC  480
             ||||||||||||||||||||||||||||||||||||||||||||||||||||||||||||
Sbjct  429   CATGAAGCCTCATTAGGGGTGAGCTCAGCATGTCCATACCAGGGAAAGTCCTCCTTTTTC  488

Query  481   AGAAATGTGGTATGGCTTATCAAAAAGAACAGTACATACCCAACAATAAAGAGGAGCTAC  540
             ||||||||||||||||||||||||||||||||||||||||||||||||||||||||||||
Sbjct  489   AGAAATGTGGTATGGCTTATCAAAAAGAACAGTACATACCCAACAATAAAGAGGAGCTAC  548

Query  541   AATAATACCAACCAAGAAGATCTTTTGGTACTGTGGGGGATTCACCATCCTAATGATGCG  600
             ||||||||||||||||||||||||||||||||||||||||||||||||||||||||||||
Sbjct  549   AATAATACCAACCAAGAAGATCTTTTGGTACTGTGGGGGATTCACCATCCTAATGATGCG  608

Query  601   GCAGAGCAGACAAAGCTCTATCAAAACCCAACCACCTATATTTCCGTTGGGACATCAACA  660
```

```
||||||||||||||||||||||||||||||||||||||||||||||||||||||||||||
Sbjct  609   GCAGAGCAGACAAAGCTCTATCAAAACCCAACCACCTATATTTCCGTTGGGACATCAACA  668

Query  661   CTAAACCAGAGATTGGTACCAAGAATAGCTACTAGATCCAAAGTAAACGGGCAAAGTGGA  720
||||||||||||||||||||||||||||||||||||||||||||||||||||||||||||
Sbjct  669   CTAAACCAGAGATTGGTACCAAGAATAGCTACTAGATCCAAAGTAAACGGGCAAAGTGGA  728

Query  721   AGGATGGAGTTCTTCTGGACAATTTTAAAACCGAATGATGCAATCAACTTCGAGAGTAAT  780
||||||||||||||||||||||||||||||||||||||||||||||||||||||||||||
Sbjct  729   AGGATGGAGTTCTTCTGGACAATTTTAAAACCGAATGATGCAATCAACTTCGAGAGTAAT  788

Query  781   GGAAATTTCATTGCTCCAGAATATGCATACAAAATTGTCAAGAAAGGGGACTCAACAATT  840
||||||||||||||||||||||||||||||||||||||||||||||||||||||||||||
Sbjct  789   GGAAATTTCATTGCTCCAGAATATGCATACAAAATTGTCAAGAAAGGGGACTCAACAATT  848

Query  841   ATGAAAAGTGAATTGGAATATGGTAACTGCAACACCAAGTGTCAAACTCCAATGGGGGCG  900
||||||||||||||||||||||||||||||||||||||||||||||||||||||||||||
Sbjct  849   ATGAAAAGTGAATTGGAATATGGTAACTGCAACACCAAGTGTCAAACTCCAATGGGGGCG  908

Query  901   ATAAACTCTAGTATGCCATTCCACAATATACACCCTCTCACCATCGGGGAATGCCCCAAA  960
||||||||||||||||||||||||||||||||||||||||||||||||||||||||||||
Sbjct  909   ATAAACTCTAGTATGCCATTCCACAATATACACCCTCTCACCATCGGGGAATGCCCCAAA  968

Query  961   TATGTGAAATCAAACAGATTAGTCCTTGCGACTGGGCTCAGAAATAGCCCTCAAAGAGAG 1020
||||||||||||||||||||||||||||||||||||||||||||||||||||||||||||
Sbjct  969   TATGTGAAATCAAACAGATTAGTCCTTGCGACTGGGCTCAGAAATAGCCCTCAAAGAGAG 1028

Query  1021  AGAAGAAGAAAAAAGAGAGGATTATTTGGAGCTATAGCAGGTTTTATAGAGGGAGGATGG 1080
||||||||||||||||||||||||||||||||||||||||||||||||||||||||||||
Sbjct  1029  AGAAGAAGAAAAAAGAGAGGATTATTTGGAGCTATAGCAGGTTTTATAGAGGGAGGATGG 1088
```

```
Query  1081  CAGGGAATGGTAGATGGTTGGTATGGGTACCACCATAGCAATGAGCAGGGGAGTGGGTAC 1140
             ||||||||||||||||||||||||||||||||||||||||||||||||||||||||||||
Sbjct  1089  CAGGGAATGGTAGATGGTTGGTATGGGTACCACCATAGCAATGAGCAGGGGAGTGGGTAC 1148

Query  1141  GCTGCAGACAAAGAATCCACTCAAAAGGCAATAGATGGAGTCACCAATAAGGTCAACTCG 1200
             ||||||||||||||||||||||||||||||||||||||||||||||||||||||||||||
Sbjct  1149  GCTGCAGACAAAGAATCCACTCAAAAGGCAATAGATGGAGTCACCAATAAGGTCAACTCG 1208

Query  1201  ATCATTGACAAAATGAACACTCAGTTTGAGGCCGTTGGAAGGGAATTTAACAACTTAGAA 1260
             ||||||||||||||||||||||||||||||||||||||||||||||||||||||||||||
Sbjct  1209  ATCATTGACAAAATGAACACTCAGTTTGAGGCCGTTGGAAGGGAATTTAACAACTTAGAA 1268

Query  1261  AGGAGAATAGAGAATTTAAACAAGAAGATGGAAGACGGGTTCCTAGATGTCTGGACTTAT 1320
             ||||||||||||||||||||||||||||||||||||||||||||||||||||||||||||
Sbjct  1269  AGGAGAATAGAGAATTTAAACAAGAAGATGGAAGACGGGTTCCTAGATGTCTGGACTTAT 1328

Query  1321  AATGCTGAACTTCTGGTTCTCATGGAAAATGAGAGAACTCTAGACTTTCATGACTCAAAT 1380
             ||||||||||||||||||||||||||||||||||||||||||||||||||||||||||||
Sbjct  1329  AATGCTGAACTTCTGGTTCTCATGGAAAATGAGAGAACTCTAGACTTTCATGACTCAAAT 1388

Query  1381  GTCAAGAACCTTTACGACAAGGTCCGACTACAGCTTAGGGATAATGCAAAGGAACTGGGT 1440
             ||||||||||||||||||||||||||||||||||||||||||||||||||||||||||||
Sbjct  1389  GTCAAGAACCTTTACGACAAGGTCCGACTACAGCTTAGGGATAATGCAAAGGAACTGGGT 1448

Query  1441  AACGGTTGTTTCGAGTTCTATCATAAATGTGATAATGAATGTATGGAAAGTGTAAGAAAC 1500
             ||||||||||||||||||||||||||||||||||||||||||||||||||||||||||||
Sbjct  1449  AACGGTTGTTTCGAGTTCTATCATAAATGTGATAATGAATGTATGGAAAGTGTAAGAAAC 1508

Query  1501  GGAACGTATGACTACCCGCAGTATTCAGAAGAAGCAAGACTAAAAAGAGAGGAAATAAGT 1560
```

```
||||||||||||||||||||||||||||||||||||||||||||||||||||||||||||

Sbjct  1509  GGAACGTATGACTACCCGCAGTATTCAGAAGAAGCAAGACTAAAAAGAGAGGAAATAAGT 1568


Query  1561  GGAGTAAAATTGGAATCAATAGGAATTTACCAAATACTGTCAATTTATTCTACAGTGGCG 1620

||||||||||||||||||||||||||||||||||||||||||||||||||||||||||||

Sbjct  1569  GGAGTAAAATTGGAATCAATAGGAATTTACCAAATACTGTCAATTTATTCTACAGTGGCG 1628


Query  1621  AGTTCCCTAGCACTGGCAATCATGGTAGCTGGTCTATCCTTATGGATGTGCTCCAATGGG 1680

 ||||||||||||||||||||||||||||||||||||||||||||||||||||||||||||

Sbjct  1629  AGTTCCCTAGCACTGGCAATCATGGTAGCTGGTCTATCCTTATGGATGTGCTCCAATGGG 1688


Query  1681  TCGTTACAATGCAGAATTTGCA  1702

||||||||||||||||||||||

Sbjct  1689  TCGTTACAATGCAGAATTTGCA  1710
```

Figure S8. Alignment analysis on HA amino acid of avain influenza H5N1 viruses

```
                                        10        20        30        40        50
HA(A/Anhui/2/2005(H5N1)         MEKIVLLLGIVSLVKSDQICIGYHANNSTEQVDTIMEKNVTVTHAQDILE
HA(A/duck/Guangdong/23/2004(H5  MEKIVLLLAIVSLVKSDQICIGYHANNSTEQVDTIMEKNVTVTHAQDILE
HA(A/Thailand/Kan353/2004(H5N1  MEKIVLLFAIVSLVKSDQICIGYHANNSTEQVDTIMEKNVTVTHAQDILE
HA(A/Viet Nam/1203/2004(H5N1))  MEKIVLLFAIVSLVKSDQICIGYHANNSTEQVDTIMEKNVTVTHAQDILE
HA(A/Bar-headed Goose/Qinghai/  MEKIVLLLAIVSLVKSDQICIGYHANNSTEQVDTIMEKNVTVTHAQDILE
HA(A/Hong Kong/156/97(H5N1))    MEKTVLLLATVSLVKSDQICIGYHANNSTEQVDTIMEKNVTVTHAQDILE
Clustal Consensus               *** ***:. ****************************************

                                        60        70        80        90       100
HA(A/Anhui/2/2005(H5N1)         KTHNGKLCDLDGVKPLILRDCSVAGWLLGNPMCDEFINVPEWSYIVEKAN
HA(A/duck/Guangdong/23/2004(H5  KTHNGKLCDLDGVKPLILRDCSVAGWLLGNPMCDEFINVPEWSYIVEKAN
HA(A/Thailand/Kan353/2004(H5N1  KTHNGKLCDLDGVKPLILRDCSVAGWLLGNPMCDEFINVPEWSYIVEKAN
HA(A/Viet Nam/1203/2004(H5N1))  KKHNGKLCDLDGVKPLILRDCSVAGWLLGNPMCDEFINVPEWSYIVEKAN
HA(A/Bar-headed Goose/Qinghai/  KTHNGKLCDLDGVKPLILRDCSVAGWLLGNPMCDEFLNVPEWSYIVEKIN
HA(A/Hong Kong/156/97(H5N1))    RTHNGKLCDLNGVKPLILRDCSVAGWLLGNPMCDEFINVPEWSYIVEKAS
Clustal Consensus               :.********:*************************:*********** .

                                       110       120       130       140       150
HA(A/Anhui/2/2005(H5N1)         PANDLCYPGNFNDYEELKHLLSRINHFEKIQIIPKSSWSDHEASSGVSSA
HA(A/duck/Guangdong/23/2004(H5  PANDLCYPGNINDYEELKHLLSRINHFEKIQIIPKSSWSDHEASLGVSSA
HA(A/Thailand/Kan353/2004(H5N1  PVNDLCYPGDFNDYEELKHLLSRINHFEKIQIIPKSSWSSHEASLGVSSA
HA(A/Viet Nam/1203/2004(H5N1))  PVNDLCYPGDFNDYEELKHLLSRINHFEKIQIIPKSSWSSHEASLGVSSA
HA(A/Bar-headed Goose/Qinghai/  PANDLCYPGNFNDYEELKHLLSRINHFEKIQIIPKSSWSDHEASSGVSSA
HA(A/Hong Kong/156/97(H5N1))    PANDLCYPGNFNDYEELKHLLSRINHFEKIQIIPKSSWSNHDASSGVSSA
Clustal Consensus               *.*******::****************************.*:** *****

                                       160       170       180       190       200
HA(A/Anhui/2/2005(H5N1)         CPYQGTPSFFRNVVWLIKKNNTYPPIKRSYNNTNQEDLLILWGIHHSNDA
HA(A/duck/Guangdong/23/2004(H5  CPYQGSSSFFRNVVWLIKKNNAYPTIKRSYNNTNQENLLVLWGIHHPNDE
HA(A/Thailand/Kan353/2004(H5N1  CPYQGKSSFFRNVVWLIKKNSTYPTIKRSYNNTNQEDLLVLWGIHHPNDA
HA(A/Viet Nam/1203/2004(H5N1))  CPYQGKSSFFRNVVWLIKKNSTYPTIKRSYNNTNQEDLLVLWGIHHPNDA
HA(A/Bar-headed Goose/Qinghai/  CPYQGRSSFFRNVVWLIKKNNAYPTIKRSYNNTNQEDLLVLWGIHHPNDA
HA(A/Hong Kong/156/97(H5N1))    CPYLGRSSFFRNVVWLIKKNSAYPTIKRSYNNTNQEDLLVLWGIHHPNDA
Clustal Consensus               *** * .*************.:**.***********:**:******.** 

                                       210       220       230       240       250
HA(A/Anhui/2/2005(H5N1)         AEQTKLYQNPTTYISVGTSTLNQRLVPKIATRSKVNGRSGRMDFFWTILK
HA(A/duck/Guangdong/23/2004(H5  AEQTRLYQNPTTYISVGTSTLNQRLVPKIATRSKVNGQSGRIDFFWTILK
HA(A/Thailand/Kan353/2004(H5N1  AEQTKLYQNPTTYISVGTSTLNQRLVPRIATRSKVNGQSGRMEFFWTILK
HA(A/Viet Nam/1203/2004(H5N1))  AEQTKLYQNPTTYISVGTSTLNQRLVPRIATRSKVNGQSGRMEFFWTILK
HA(A/Bar-headed Goose/Qinghai/  AEQTRLYQNPTTYISVGTSTLNQRLVPKIATRSKVNGQSGRMEFFWTILK
HA(A/Hong Kong/156/97(H5N1))    AEQTKLYQNPTTYISVGTSTLNQRLVPEIATRPKVNGQSGRMEFFWTILK
Clustal Consensus               ****:**********************.****.****:***::*******

                                       260       270       280       290       300
HA(A/Anhui/2/2005(H5N1)         PNDAINFESNGNFIAPEYAYKIVKKGDSAIMKSEVEYGNCNTKCQTPIGA
HA(A/duck/Guangdong/23/2004(H5  PNDALNFESNGNFIAPEYAYKIVKKGDSAIMKSEVEYGNCNTKCQTPMGA
HA(A/Thailand/Kan353/2004(H5N1  PNDAINFESNGNFIAPEYAYKIVKKGDSTIMKSELEYGNCNTKCQTPMGA
HA(A/Viet Nam/1203/2004(H5N1))  PNDAINFESNGNFIAPEYAYKIVKKGDSTIMKSELEYGNCNTKCQTPMGA
HA(A/Bar-headed Goose/Qinghai/  PNDAINFESNGNFIAPENAYKIVKKGDSTIMKSELEYGNCNTKCQTPIGA
HA(A/Hong Kong/156/97(H5N1))    PNDAINFESNGNFIAPEYAYKIVKKGDSTIMKSELEYGNCNTKCQTPMGA
Clustal Consensus               ****:************ **********.*****:************:**

                                       310       320       330       340       350
HA(A/Anhui/2/2005(H5N1)         INSSMPFHNIHPLTIGECPKYVKSNKLVLATGLRNSPLRERRRK-RGLFG
HA(A/duck/Guangdong/23/2004(H5  INSSMPFHNIHPLTIGECPKYVKSNKLVLATGLRNSPQRERRRKKRGLFG
HA(A/Thailand/Kan353/2004(H5N1  INSSMPFHNIHPLTIGECPKYVKSNRLVLATGLRNSPQRERRRKKRGLFG
HA(A/Viet Nam/1203/2004(H5N1))  INSSMPFHNIHPLTIGECPKYVKSNRLVLATGLRNSPQRERRRKKRGLFG
HA(A/Bar-headed Goose/Qinghai/  INSSMPFHNIHPLTIGECPKYVKSNRLVLATGLRNSPQGERRRKKRGLFG
HA(A/Hong Kong/156/97(H5N1))    INSSMPFHNIHPLTIGECPKYVKSNRLVLATGLRNTPQRERRRKKRGLFG
Clustal Consensus               *************************:*********:*  ***** *****

                                       360       370       380       390       400
HA(A/Anhui/2/2005(H5N1)         AIAGFIEGGWQGMVDGWYGYHHSNEQGSGYAADKESTQKAIDGVTNKVNS
HA(A/duck/Guangdong/23/2004(H5  AIAGFIEGGWQGMVDGWYGYHHSNEQGSGYPADKESTQKAIDGVTNKVNS
HA(A/Thailand/Kan353/2004(H5N1  AIAGFIEGGWQGMVDGWYGYHHSNEQGSGYAADKESTQKAIDGVTNKVNS
HA(A/Viet Nam/1203/2004(H5N1))  AIAGFIEGGWQGMVDGWYGYHHSNEQGSGYAADKESTQKAIDGVTNKVNS
HA(A/Bar-headed Goose/Qinghai/  AIAGFIEGGWQGMVDGWYGYHHSNEQGSGYAADKESTQKAIDGVTNKVNS
HA(A/Hong Kong/156/97(H5N1))    AIAGFIEGGWQGMVDGWYGYHHSNEQGSGYAADKESTQKAIDGVTNKVNS
Clustal Consensus               ******************************.*******************
```

```
                                      410       420       430       440       450
HA(A/Anhui/2/2005(H5N1)               IIDKMNTQFEAVGREFNNLERRIENLNKKMEDGFLDVWTYNAELLVLMEN
HA(A/duck/Guangdong/23/2004(H5        IIDKMNTQFEAVGREFNNLERRIENLNKKMEDGFLNVWTYNAELLVLMEN
HA(A/Thailand/Kan353/2004(H5N1        IIDKMNTQFEAVGREFNNLERRIENLNKKMEDGFLDVWTYNAELLVLMEN
HA(A/Viet Nam/1203/2004(H5N1))        IIDKMNTQFEAVGREFNNLERRIENLNKKMEDGFLDVWTYNAELLVLMEN
HA(A/Bar-headed Goose/Qinghai/        IIDKMNTQFEAVGREFNNLERRIENLNKKMEDGFLDVWTYNAELLVLMEN
HA(A/Hong Kong/156/97(H5N1))          IINKMNTQFEAVGREFNNLERRIENLNKKMEDGFLDVWTYNAELLVLMEN
Clustal Consensus                     **:********************************:**************

                                      460       470       480       490       500
HA(A/Anhui/2/2005(H5N1)               ERTLDFHDSNVKNLYDKVRLQLRDNAKELGNGCFEFYHKCDNECMESVRN
HA(A/duck/Guangdong/23/2004(H5        ERTLNFHDSNVKNLYDKVRLQLRDNAKELGNGCFEFYHKCDNECMESVKN
HA(A/Thailand/Kan353/2004(H5N1        ERTLDFHDSNVKNLYDKVRLQLRDNAKELGNGCFEFYHKCDNECMESVRN
HA(A/Viet Nam/1203/2004(H5N1))        ERTLDFHDSNVKNLYDKVRLQLRDNAKELGNGCFEFYHKCDNECMESVRN
HA(A/Bar-headed Goose/Qinghai/        ERTLDFHDSNVKNLYDKVRLQLRDNAKELGNGCFEFYHRCDNECMESVRN
HA(A/Hong Kong/156/97(H5N1))          ERTLDFHDSNVKNLYDKVRLQLRDNAKELGNGCFEFYHKCDNECMESVKN
Clustal Consensus                     ****:*********************************:*********:*

                                      510       520       530       540       550
HA(A/Anhui/2/2005(H5N1)               GTYDYPQYSEEARLKREEISGVKLESIGTYQILSIYSTVASSLALAIMVA
HA(A/duck/Guangdong/23/2004(H5        GTYDYPQYSEEARLKREEISGVKLESIGTYQILSIYSTVASYLALAIMVA
HA(A/Thailand/Kan353/2004(H5N1        GTYDYPQYSEEARLKREEISGVKLESIGIYQILSIYSTVASSLALAIMVA
HA(A/Viet Nam/1203/2004(H5N1))        GTYDYPQYSEEARLKREEISGVKLESIGIYQILSIYSTVASSLALAIMVA
HA(A/Bar-headed Goose/Qinghai/        GTYDYPQYSEEARLKREEISGVKLESIGTYQILSIYSTVASSLALAIMVA
HA(A/Hong Kong/156/97(H5N1))          GTYDYPQYSEEARLNREEISGVKLESMGTYQILSIYSTVASSLALAIMVA
Clustal Consensus                     **************:***********:* ************ ********

                                      560
HA(A/Anhui/2/2005(H5N1)               GLSLWMCSNGSLQCRICI
HA(A/duck/Guangdong/23/2004(H5        GLSLWMCSNGSLQCRICI
HA(A/Thailand/Kan353/2004(H5N1        GLSLWMCSNGSLQCRIC-
HA(A/Viet Nam/1203/2004(H5N1))        GLSLWMCSNGSLQCRICI
HA(A/Bar-headed Goose/Qinghai/        GLSLWMCSNGSLQCRIA-
HA(A/Hong Kong/156/97(H5N1))          GLSLWMCSNGSLQCRICI
Clustal Consensus                     **************** .
```
